# Molecular Mechanisms and Epigenetic Regulation in Diabetic Cardiomyopathy

**DOI:** 10.3389/fcvm.2021.725532

**Published:** 2021-12-16

**Authors:** Anupam Mittal, Rajni Garg, Ajay Bahl, Madhu Khullar

**Affiliations:** ^1^Department of Translational and Regenerative Medicine, Postgraduate Institute of Medical Education and Research, Chandigarh, India; ^2^Council of Scientific and Industrial Research - Institute of Microbial Technology, Chandigarh, India; ^3^Department of Cardiology, Postgraduate Institute of Medical Education and Research, Chandigarh, India; ^4^Department of Experimental Medicine and Biotechnology, Postgraduate Institute of Medical Education and Research, Chandigarh, India

**Keywords:** diabetes mellitus, diabetic cardiomyopathy, apoptosis, oxidative stress, mitochondrial function, cardiac remodeling, epigenetics

## Abstract

Diabetes mellitus (DM) is an important lifestyle disease. Type 2 diabetes is one of the prime contributors to cardiovascular diseases (CVD) and diabetic cardiomyopathy (DbCM) and leads to increased morbidity and mortality in patients with DM. DbCM is a typical cardiac disease, characterized by cardiac remodeling in the presence of DM and in the absence of other comorbidities such as hypertension, valvular diseases, and coronary artery disease. DbCM is associated with defective cardiac metabolism, altered mitochondrial structure and function, and other physiological and pathophysiological signaling mechanisms such as oxidative stress, inflammation, myocardial apoptosis, and autophagy. Epigenetic modifiers are crucial players in the pathogenesis of DbCM. Thus, it is important to explore the role of epigenetic modifiers or modifications in regulating molecular pathways associated with DbCM. In this review, we have discussed the role of various epigenetic mechanisms such as histone modifications (acetylation and methylation), DNA methylation and non-coding RNAs in modulating molecular pathways involved in the pathophysiology of the DbCM.

## Introduction

Diabetic cardiomyopathy (DbCM) is a cardiac disease characterized by functional and structural abnormalities in cardiac tissue in patients having diabetes mellitus (DM) but no other comorbidities such as hypertension, valvular diseases, and coronary artery disease (1). Framingham Heart Study observed that women and men with DM have 5- and 2.4-fold higher incidence of heart failure (HF), respectively (2). Patients with diabetes have a high prevalence of HF ranging from 19 to 26% (3–5). A case-control study found that the prevalence of HF was 1.3 times higher in diabetic subjects in comparison with the non-diabetic subjects (6). In both type I diabetes (T1D) and type II diabetes (T2D), patients showed a strong correlation between glycated hemoglobin A(1c) (HbA1c) and HF. With every 1% increase in HbA1c, there is a 30 and 8% higher incidence of HF in T1D and T2D, respectively, independent of other risk factors (7, 8). The initial phase of DbCM is characterized by extensive cardiac hypertrophy and mild to moderate fibrosis, leading to defects in the systolic and diastolic function of the heart (9).

**Figure 1:**
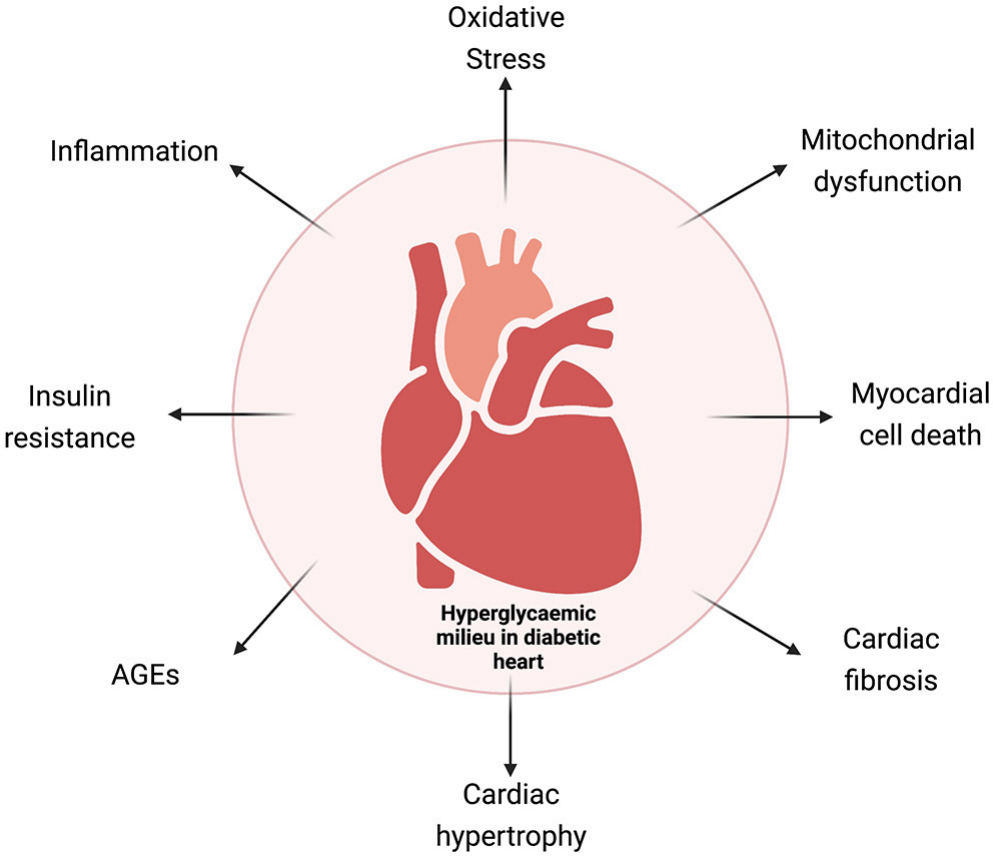
Schematic representation of the various mechanisms involved in diabetic cardiomyopathy. AGEs, advanced glycation end products.

Experimental and clinical studies have identified sustained hyperglycemia (HG), insulin resistance, aberrant insulin signaling, impaired glucose metabolism, abnormal free fatty acid (FFA) uptake, oxidative stress, increased renin–angiotensin–aldosterone (RAAS) activity, cardiac inflammation, and aberrant mitochondrial function as the key determinants for biochemical alterations leading to a vicious cycle of disease. Cardiac fibrosis, left ventricular (LV) hypertrophy, and increased cardiomyocyte cell death are the most important mechanisms to explain the pathophysiology of the disease (10, 11). Multiple molecular mechanisms have been identified contributing to pathophysiological changes in DbCM (Figure 1), which include O-GlcNAcylation of cardiac proteins, decreased insulin and AMPK signaling, activated MAPK, peroxisome proliferator-activated receptors, and aberrant protein kinase C activity.

Recent studies suggest that epigenetic regulatory mechanisms such as DNA methylation, histone modifications (acetylation and methylation), deregulated microRNAs (miRNAs), circular RNA (circRNAs), and long non-coding RNA (lncRNAs) play an important role in the pathogenesis of DbCM (12, 13). In this review, we provide a comprehensive overview of the role of epigenetic modifications in various molecular pathways associated with DbCM.

## Molecular Mechanisms and their Epigenetic Regulations in DbCM

### Cardiac Remodeling in DbCM

Cardiomyocyte hypertrophy and fibrosis are the important features of DbCM. Cardiac fibrosis is a dominant mechanism contributing to the disease pathology of the diabetic human heart. There is a very pronounced deposition of collagen in interstitial and perivascular spaces in diabetic cardiac tissues (14). The major contributing pathways for the aggravated deposition of collagen types I and III are transforming growth factor-β1 (TGF-β1) and wingless-related integration site (WNT) signaling pathways (15). Additionally, there is remodeling of matrix metalloproteinases (MMPs) leading to dysregulated degradation of extracellular matrix in diabetic hearts (15–18). Activation of the TGF-β1 pathway and accelerated extracellular matrix degradation are mainly consequences of stimulation of RAAS resulting in heightened advanced glycation end products (AGEs)-mediated signaling, HG, and insulin resistance (19). Decreased availability of nitric oxide (NO), oxidative stress, activation of TGF-β1 signaling pathway, in association with deregulated insulin signaling leads to high cardiac collagen deposition and fibronectin content, leading to interstitial fibrosis (20). Several clinical and animal studies provide substantial evidence of cardiac fibrosis in diabetes-induced heart failure (HF) (18, 21–23).

An increase in LV hypertrophy represented by high LV mass and its association with DM is well-established (24–26). Thickened LV is a major hallmark of cardiac hypertrophy in humans (27). Cardiac fibrosis, hypertrophy, and myocardial cell apoptosis must be taken into account for the overall increase in LV mass (27, 28). In DbCM, there are other contributors in addition to cardiac hypertrophy such as insulin resistance, HG in the milieu, and oxidative stress-activating cardiac hypertrophic genes, such as β-myosin heavy chain (ß-MHC), atrial natriuretic factor (ANP), and brain natriuretic factor (BNP) (29). Heightened insulin levels induce cardiac hypertrophy. Insulin-like growth factor (IGF-1) induces cardiomyocyte hypertrophy through activation of the mitogen-activated protein kinase 1 (Erk1/2) and phosphoinositide 3-kinases (PI3K) signaling pathways (30). Several studies in animal models of DbCM have also shown the role of DM in the development of cardiac or cardiomyocyte hypertrophy (31–33).

#### Epigenetic Regulation of Cardiac Remodeling in DbCM

MicroRNAs are small, non-coding RNAs, which regulate cellular gene expression. Aberrant expression of ~30% miRNAs (that is 300 out of 1,000 total miRNAs) has been observed in DM heart tissues (34). Several miRNAs have been found to regulate cardiac fibrosis and cardiac hypertrophy in DbCM. For example, miRNA-221 was shown to be highly upregulated in the cardiac tissue of diabetic mice (35). miRNA-212 was found to regulate the process of cardiac hypertrophy by directly regulating Forkhead box O3 (Foxo3) (35). Raut et al. reported that miRNA-30c mediates increased expression of hypertrophy genes, cell division control protein 42 homolog (Cdc42), and Rac1-activated kinase 1 (Pak1) in DbCM (36). Other miRNAs, such as 181a and 200c, were shown to play a pivotal role in cardiac remodeling (35, 37–39). The expression of miRNA-199a was elevated in cardiac hypertrophy (34). Recently, it was reported that silencing of miR-199a led to the reversal of cardiac hypertrophy by rescuing the mitochondrial fatty acid oxidation through targeting peroxisome proliferator-activated receptor-gamma coactivator (PGC-1α) (40). miRNA-30a, miRNA-1, and miRNA-29b levels were found to be downregulated in the diabetic heart (34). miRNA-144 and miRNA-133a are among the important key players, involved in the pathophysiology of diabetes-mediated HF (41, 42). Singh et al. showed that miRNA-200c promoted cardiac hypertrophy by modulating dual-specific phosphatase 1 (DUSP1) expression in DbCM (39).

Decreased levels of miRNA-133a were observed in the diabetic murine model (43). It was also seen that increase in miRNA-133a levels improved the systolic function and reduced fibrosis by decreasing the collagen (44). miRNA-21 has been established as a biomarker for cardiac fibrosis (45). Several groups including ours have shown upregulated miRNA-21 levels in rat cardiac fibroblasts in the hyperglycemic milieu and in diabetic hearts, which leads to advanced collagen synthesis and fibroblast proliferation (46, 47). miRNA-21 was also found to directly regulate dual-specific phosphatase 8 (DUSP8) by perturbing c-Jun N-terminal kinase (JNK) and p38 MAP kinase (MAPK) signaling pathways (46).

Long non-coding RNAs, a type of non-coding RNAs which are longer than miRNAs, have been implicated in various disease pathways (48). Nuclear lncRNAs act at the transcriptional level, and cytoplasmic lncRNAs often interact with miRNAs to regulate gene expression post-transcriptionally. Several lncRNAs have been recently shown to be involved in the pathophysiology of CVDs, including DbCM, contributing to cardiac hypertrophy and fibrosis. Myocardial infarction-associated transcript (MIAT) acts as prohypertrophic lncRNA as it has a sponging activity for antihypertrophic miRNAs, miRNA-150 (49), and miRNA-93 (50). Additionally, MIAT levels were higher in the myocardium and compete with miRNA-24 levels to regulate TGF-β1 expression and thus cardiac fibrosis (51). LncRNA Kcnq1ot1 ablation ameliorates TGF-β1 signaling and, thus, reduces fibrotic lesions in diabetic mice (52). The dysregulated ncRNAs, both miRNA and lncRNA, explain HG-related myocardial insult.

Histone modifications also have been found to play a crucial role in the cardiac remodeling in DbCM. Non-specific inhibitor-based silencing of histone deacetylases (HDACs) has been shown to attenuate cardiac hypertrophy and fibrosis, by increasing the glucose transporter 1 acetylation and MAPK-mediated phosphorylation in animal models of diabetic heart disease (53). The use of specific HDAC3 inhibitors such as RGFP966 also showed improved cardiac function and reversed DM-induced cardiac remodeling in diabetic mice. It was found that RGFP966 decreased cardiac hypertrophy by epigenetic modulation of the ERK1/2 pathway mediated by DUSP5 (54). In contrast, Sir2 is known to have a beneficial effect on DCM. It improves contractile dysfunction in leptin receptor-deficient db/db mice through a histone deacetylase Sir2-driven pathway (54), suggesting its potential as a therapeutic molecule in DbCM. (55).

### Role of Epigenetics in Regulating Cell Death Mechanisms in DbCM

Diabetic cardiomyopathy has a strong association with high cardiomyocyte cell death. Apoptosis and autophagy are the important deregulated mechanisms responsible for this phenomenon (56). Several fold higher apoptosis rates have been reported in cardiomyocytes, fibroblasts, and endothelial cells in myocardial tissues of patients with DbCM. The death rate of cardiomyocytes was the highest, followed by that of endothelial cells and fibroblasts (57). Increased cardiomyocyte cell death results in cell loss in the heart, remodeling such as cardiac hypertrophy and fibrosis, leading to cardiomyopathy and cardiac failure (58).

Various mechanisms are proposed for increased cardiomyocyte cell death in diabetic hearts. HG, insulin resistance, lipid peroxidation, increased angiotensin II signaling, oxidative stress, and endoplasmic stress have been implicated as major triggers of cardiomyocyte apoptosis in the diabetic milieu. HG is the major causative factor for increased oxidative stress and endoplasmic stress mediating cardiomyocyte death in diabetic hearts (59). HG mediates these actions through localized increased angiotensin II (Ang II) (60). Kobayashi et al. have recently shown that HG may also induce cardiomyocyte cell death by inducing lysosomal membrane permeabilization and increased cathepsin D expression and lysosomal release in cardiomyocytes resulting in cell death (61).

#### Apoptosis and Its Epigenetic Regulation

The expression of several miRNAs was deregulated in HG-induced cardiomyocyte apoptosis and diabetic hearts (34). These include miRNA-30c, miRNA-181, miRNA-378, miRNA-34a, miRNA-1, miRNA-195, miRNA-144, and miRNA-483-3p. It was shown that miR-1 is upregulated in HG-treated H9c2 cardiomyocytes along with increased apoptosis. They reported that miR-1 promotes cardiomyocyte apoptosis by inhibiting IGF-1 expression; IGF-1 increased expression was shown to inhibit glucose-induced cytochrome c release and apoptosis, suggesting that miRNA-1 promotes apoptosis by regulating IGF-1 (62). miRNA-34a is highly expressed in cardiomyocytes and regulates the expression of several proteins including prosurvival protein, sirtuin 1 (SIRT1). miRNA-34a is upregulated in diabetic hearts and glucose-treated cardiomyocytes. Fomison-Nurse et al. reported that upregulation of miRNA-34a was associated with downregulation of SIRT1 and increased the activity of proapoptotic caspases in HG-treated cultured cardiomyocytes. Inhibition of miRNA-34a was found to reduce HG-induced cardiomyocyte apoptosis, indicating its potential therapeutic role (63). Qiao et al. showed that miRNA-483-3p was involved in HG-induced cardiomyocyte apoptosis by repressing the expression of its target gene, IGF-1. They reported elevated expression of this miRNA in diabetic mice and hyperglycemic cardiomyocytes (64). Downregulation of miRNA-30c and miRNA-181 was observed in diabetic hearts and hyperglycemic cardiomyocytes (38). These miRNAs promote cardiomyocyte apoptosis by deregulation of a p53-p21 axis (38). It was reported that miRNA-195 upregulation induces apoptosis in streptozotocin (STZ) and leptin receptor-deficient type 2 diabetic murine hearts *via* downregulation of SIRT1 and B cell leukemia 2 (Bcl2) (65). Altered expression of miRNA-144 was observed in hearts and also cardiomyocytes in hyperglycemic conditions. miRNA-144-3p was found to be upregulated in T2D (66). Karolina et al. reported that miRNA-144 controls the expression of IRS-1 in diabetes (66). Recently, Song et al. have reported increased miRNA-144 levels in HG-treated cardiomyocytes (67). They showed that miRNA-144-targeted C1q/TNF-related protein 3 (CTRP3)/JNK pathway and inhibition of miRNA-144 attenuated cardiomyocyte apoptosis. In another study, Tao et al. observed decreased miRNA-144 levels in HG-treated cardiomyocytes and diabetic hearts (68). Cellular overexpression of miRNA-144 resulted in improved mitochondrial function and decreased myocyte apoptosis by regulating Rac family small GTPase 1 (Rac-1) levels, which in turn regulated apoptosis *via* 5' AMP-activated protein kinase (AMPK) phosphorylation and PGC-1α deacetylation (68). Thus, the precise role of this miRNA remains to be fully elucidated. Altered PI3K/Akt signaling stimulates apoptosis, fibrosis, and hypertrophy of cardiomyocytes and leads to DbCM progression (69). It was reported that miRNA-203 overexpression inhibited PIK3CA and activated of PI3K/Akt signaling, thus inhibiting myocardial hypertrophy, fibrosis, and apoptosis (69). Recently, miRNA-532 has been shown to exhibit a positive association with cardiomyocyte apoptosis in diabetic heart disease. miRNA-532 was shown to be upregulated in cardiac tissues of patients with type 2 DM, thus decreasing the expression of its main target, the antiapoptotic protein (ARC). It was shown that miRNA-532 upregulation leads to the activation of proapoptotic caspases activity and vice versa in HG-treated cardiomyocytes (70). Another study showed decrease in expression of antiapoptotic protein, Hsp60 in the diabetic heart. miRNA-1 and miRNA-206 modulated myocardial Hsp60 post-transcriptionally and its downregulation was an important proapoptotic signal in the diabetic myocardium (71).

Besides miRNAs, several lncRNAs have been identified mediating cardiac cell death in hyperglycemic or diabetic conditions (72). Decreased expression of lncRNA H19 in DCM and HG-treated cardiomyocytes was observed and improved ventricular function by inhibiting reduced apoptosis in diabetic rats (73). H19 functions by inhibiting miR-675-mediated expression of voltage-dependent anion channel 1 (VDAC1), a proapoptotic molecule, which promotes cell death (73). Yang et al. showed escalated expression of another lncRNA Kcnq1ot, in the hearts of diabetic mice. They further showed that inhibition of Kcnq1ot1 improved cardiac function and attenuated pyroptosis (52). Metastasis-associated lung adenocarcinoma transcript 1 (MALAT1) is another long non-coding RNA that regulates HG-induced cardiomyocyte apoptosis (74). MALAT1 was also shown to downregulate miR-141 or miR-181a-5p levels by sponging and inducing NLR family pyrin domain containing 3 (NLRP3) inflammasome activity and TGF-β1/Smad signaling (75). In a very recent study, MALAT1 has been shown to influence cardiomyocyte apoptosis by EZH2, a histone methyltransferase, and upregulating ATP-binding cassette transporter A1 (ABCA1) (76). HOTAIR is another lncRNA that has been shown to protect cardiac cell death in hyperglycemic conditions and DbCM. HOTAIR was decreased in the hearts of the diabetic mice, and its cardiac-specific overexpression attenuated cardiomyocyte death in STZ diabetic mice (77). It was shown to regulate miR-34a levels by acting as competing endogenous RNA (ceRNA) and increasing its target protein SIRT1, which has antiapoptotic activity (77). MEG3 is a lncRNA that is upregulated in HG-treated cardiomyocytes and induces apoptosis *via* sponging miR-145 and increasing proapoptotic programmed cell death 4 (PDCD4) levels (78). Recently, expression of Lnc NKILA (nuclear factor-κ B interacting long non-coding RNA) was found to be highly increased in patients with DbCM and its *in vitro* silencing decreased HG-induced cardiac cell death (79). Similarly, increased lncRNA LUCAT1 (lung cancer-associated transcript 1) levels were found in HG-treated AC 16 cardiomyocytes and its inhibition reduced HG-induced cardiomyocyte apoptosis by downregulating aldosterone synthase (CYP11B2) (80).

The DNA and histone methylation and acetylation are important epigenetic mechanisms that regulate gene expression and associated cellular mechanisms. The role of these mechanisms in diabetic cardiomyocyte cell death has not been well-investigated but emerging research suggests that they might have an important role. Yu et al. reported that HDAC1 mediates repression of IGF-1R in HG-treated cardiomyocytes (81). They showed that the association of histone 4 with p53-HDAC1 is increased and the association of histone 4 with IGF-1R is decreased (81). HDAC inhibition was later shown to inhibit HG-induced cardiac apoptosis by increasing GLUT1 acetylation and decreasing caspase 3 activity in diabetic mice (53).

Endoplasmic reticulum (ER) stress, an important mediator of DbCM, has also been implicated in the induction of apoptosis of cardiac cells (82). The role of epigenetic regulation of ER stress in DM-induced cardiac apoptosis was further confirmed by Guo et al. They reported that activation of SIRT1, a deacetylase, attenuates ER stress and apoptosis in cardiomyocytes of diabetic rats (83). Nitrosative stress induced by increased nitric oxide production resulting in nitrosylation of proteins has been found to induce apoptosis in heart of diabetic rats (84). Puthanveetil et al. reported that HC-induced iNOS expression in cardiomyocytes leads to increased nitrosylation of caspase 3 that facilitates apoptosis. They showed that nitrosylation of the proteins was mediated by Foxo1. Foxo1-mediated nitrosylation of caspase 3 resulted in increased cell death under HG conditions (84).

#### Autophagy and Its Epigenetic Regulation

Autophagy is a physiological process that removes or recycles damaged cell components such as organelles, proteins, and metabolites from the cell. It is an important process to maintain cell homeostasis. Both repression and augmentation of autophagy have been reported in diabetic hearts and HG-exposed cardiomyocytes (85–87). Mellor et al. reported increased autophagy (LC3B-II: LC3B-I ratio) in hearts of fructose-fed diabetic mice, suggesting myocardial autophagy activation in DbCM (88). However, Xie et al. reported repressed cardiac AMPK activity and autophagy in OVE26 diabetic mice (89). To date, there is no unequivocal consensus on the role of myocardial autophagy in the pathophysiology of DbCM. A recent review on autophagy in diabetic heart showed that autophagy might act as a double-edged sword, with initial activation helping in the removal of damaged mitochondria, peroxisomes, and protein aggregates and improving antioxidant mechanisms through the activation of antioxidant transcription factors such as nuclear factor erythroid 2-related factor 2 (Nrf2). However, this increased autophagy in the cell may result in self-digestion and enhanced reactive oxygen species (ROS) generation, causing cardiac damage (87).

Few reports suggest that miRNAs may regulate diabetes-induced autophagy. Chen et al. reported that circulatory miRNA-30c levels were highly reduced in patients with DM. Similar results have been found in an animal model of diabetes and cardiomyocytes. It was observed that miR-30c directly regulates Beclin-1 expression. Thus, downregulation of miR-30cenhanced autophagy by increasing proautophagic Beclin-1 expression in diabetic hearts. Further, miR-30c directly regulates Beclin-1, thus controlling autophagy in DM (90).

There are a few studies suggesting the involvement of LncRNAs in DbCM. Feng et al. showed a marked increase in expression of LncRNAs, DCM-related factor (DCRF) in DbCM in the diabetic mice model (91). They showed that DCRF increased cardiomyocyte autophagy by sponging miR-551b-5p, thereby increasing protocadherin 17 (PCDH17) expression (89). Similarly, Zhou et al. reported decreased expression of LncRNA H19 in DbCM. They observed that LncRNA H19 regulates GTP-binding protein Di-Ras3 (DIRAS3) expression and promotes mTOR phosphorylation, thus inhibiting autophagy in DbCM (92).

#### Pyroptosis and Its Epigenetic Regulation

Pyroptosis or inflammation-induced cell death has been shown to contribute to increased cardiomyocyte cell loss in DbCM (93). miRNA-30d promoted cardiomyocyte pyroptosis in hyperglycemic conditions by repressing Forkhead box O3 (Foxo3a) and its downstream effector activity regulated cytoskeleton-associated protein (ARC), an apoptotic repressor leading to caspase-1 activation and increasing proinflammatory molecules (94). Jeyabal et al. have also reported that miRNA-9 may have a role in HG-induced cardiomyocyte pyroptosis (95). They showed that expression of miRNA-9 was significantly decreased in HG-treated cardiomyocytes *in vitro* and in human diabetic hearts *in vivo*. The proinflammatory ELAV-like protein 1 (ELAVL1) was shown to be the target protein of miRNA-9. The authors reported that upregulation of miRNA-9 attenuated HG-induced cardiomyocyte pyroptosis by downregulating ELAVL1 expression, indicating that miRNA-9 has an antiapoptotic role in diabetic hearts (95).

### Epigenetic Regulation of Mitochondrial Dysfunction in DbCM

Mitochondria play a vital role in the maintenance of cardiac function and metabolism. Loss of mitochondrial function is implicated in DbCM (96). In adult cardiomyocytes, oxidative phosphorylation is the major source of intracellular ATP production in mitochondria. During DM, there is a switch in the ATP production pathway from glucose to FFA oxidation in mitochondria (97). This impaired oxidative phosphorylation increases mitochondrial ROS generation (98). Further, faulty Ca^2+^ flux in mitochondria leads to apoptosis in cardiomyocytes (99). This dysregulated Ca^2+^ flux also induces permeability in mitochondrial membranes, resulting in increased cardiomyocyte autophagy (100).

MicroRNAs have a significant role in fatty acid metabolism in the diabetic heart tissues. miRNA-133a levels were decreased in the cardiac tissue of the diabetic murine model (43). Mechanistically, miRNA-133a controls the CD36 expression by directly regulating testicular protein 4 (101). This explains the increase in CD36 expression in diabetic rat hearts (102). Peroxisome proliferator-activated receptor alpha (PPAR-α) regulates the oxidation of fatty acids in cardiomyocytes (103). miRNA-29a directly regulates the coactivator of PPAR-α (104). It was also reported that miRNA-29a levels are decreased in diabetic rat hearts, and this explains the increased fatty acid oxidation mediated by PPAR-α (105). In another study, it was reported that miRNA-210 levels are 2.5 folds higher in human diabetic failing hearts compared with non-diabetic failing hearts (106). miRNA-210 is a direct regulator of ISCU1/2, iron sulfur complex protein, which drives the electron transport chain (ETC) by regulating the function of aconitase and complex I (107). Another group reported that there is an increase in miRNA-141 levels in type 1 diabetic hearts (108). miRNA-141 regulates inorganic phosphate transport in the mitochondria by regulating the solute carrier family 25 members 3 (SLC25A3), this, in turn, affects the ATP synthesis in mitochondria (108). Similarly, miRNA-378 that negatively regulates ATP synthase was found to be elevated in interfibrillar mitochondria in streptozotocin-induced diabetic hearts of mice (109). All studies suggest that miRNAs are important players in mitochondrial function and energy metabolism in the diabetic heart.

Histone modifiers such as deacetylases and acetyltransferases regulate global acetylation levels in various physiological states of the cell. They maintain homeostasis by acetylation or deacetylation of histone substrates (110). It was reported that increased HDAC activity leads to myocardial ischemia mediated by Foxo3a/Bim in the diabetic heart (111). Well-known cardiac protector resveratrol reduces mitochondrial dysfunction through regulation of SIRT1 activation in a murine model of DM and increased histone deacetylation of PGC-1α (112, 113). In the murine model of DM, HDAC inhibition resulted in elevated expression of cardiac PPAR-α and resulted in reduced expression of peroxisome proliferator-activated receptor gamma (PPAR-γ), suggesting the role of HDAC abrogation in regulating the fatty acid oxidation in DbCM (114).

### Mitochondrial Oxidative Stress and Its Epigenetic Regulation in DbCM

**Figure 2:**
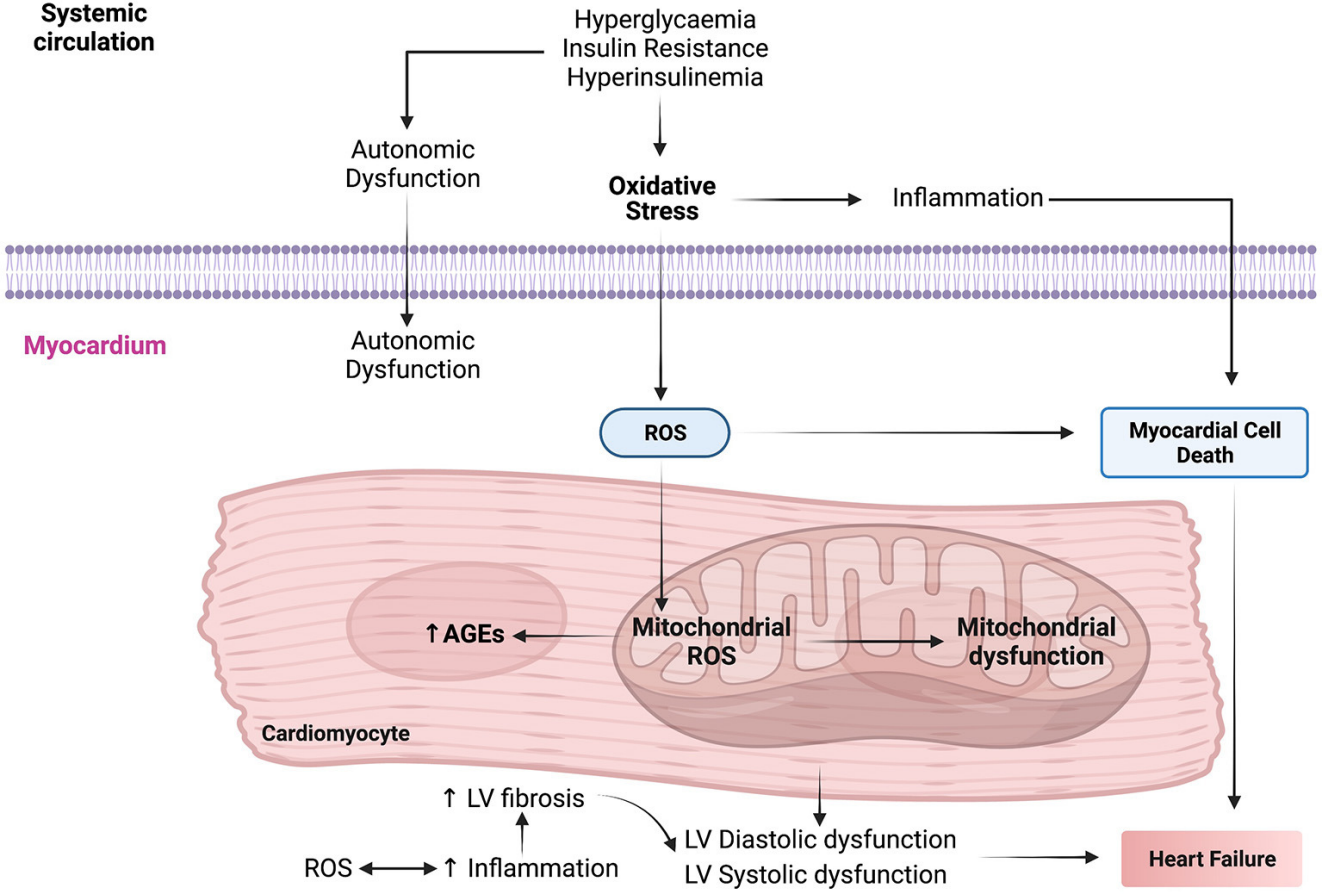
The molecular mechanisms interactome in the pathophysiology of diabetic cardiomyopathy (DbCM). AGEs, advanced glycation end products; LV, left ventricle; ROS, reactive oxygen species.

Oxidative stress plays a crucial role in the pathogenesis and progression of DbCM by increasing insulin resistance in cardiomyocytes (Figure 2). During oxygen metabolism in mitochondria, ROS is produced as a by-product (97). Under abnormal conditions such as insulin resistance and HG, there is an increase in NADH in the mitochondrial respiratory chain leading to shunting of ETC at complex III and leading to tremendous ROS production (115). High NADPH oxidase activity is seen in cardiomyocytes of patients with obesity and cardiac insulin resistance (31). This increase in NADPH oxidase activity led to increased ROS generation. In DbCM, ROS levels also get elevated because of increased xanthine oxidase activity and NO synthase uncoupling (116). Mitochondrial dysfunction leads to increased ROS accumulation. Major ROS determinants are hydrogen peroxide, hydroxyl radical, superoxide molecules, and reduced oxygen in patients with DbCM (116–118).

Several miRNAs such as miRNA-1, miRNA-19b, and miRNA-144 have been associated with oxidative stress (34, 41). It was shown that miR-1 levels decrease in cardiomyocytes treated with high glucose and treatment with N-acetylcysteine (NAC) leads to the rescue of cardiac phenotype proving the role of miRNA in oxidative stress-dependent DbCM (119). Similarly, miRNA-144 levels were found to be downregulated in hyperglycemic conditions. miRNA-144 is shown to regulate ROS levels directly through Nrf2 expression (41). Moreover, an increase in miRNA-141 in type I diabetic mice heart inhibited mitochondrial phosphate carrier (Slc25a) resulting in increased ROS and decreased mitochondrial ATP generation (108). Additionally, miRNA-210 has been reported to regulate mitochondrial metabolism by targeting the molecules involved in the ROS generation (120). Another report suggests that miRNA-373 levels were decreased in DbCM due to glucose-induced oxidative stress-mediated cardiac hypertrophy (121).

Few lncRNAs have been reported to regulate diabetes-induced oxidative stress. LncRNA H19 was shown to be downregulated in diabetic rat hearts, and enforced overexpression leads to attenuation of oxidative stress and thus, in turn, alleviates the LV dysfunction (73).

Epigenetic modulators such as histone DNA deacetylases have been also found to play an important role in oxidative stress-mediated pathophysiology of DbCM. Kumar et al. reported that dysregulated SIRT1 and methyltransferase 3b (Dnmt3b) activity resulted in increased histone H3 acetylation and CpG demethylation at the *p66Shc* (prooxidant adaptor protein) promoter in diabetes-induced vascular oxidative stress (122, 123). Similarly, Mortuza et al. looked into the mechanisms that decreased SIRT1 activity and suggested the role of SIRT1 and FOXO1 axis in ROS-mediated stress (124).

### Electrical Remodeling and Its Epigenetic Regulation in the Progression of DbCM

Structural cues such as cardiac remodeling (fibrosis and hypertrophy) lead to functional abnormality (altered electrical activation) ultimately leading to electrical remodeling of the heart during HF. Electrical remodeling is characterized by compensatory or maladaptive prolonged disturbances in ion channels that might be reversible or irreversible, respectively. The remodeling of the electrical conduction system is considered to be the main reason for lethal arrhythmias (125). There are various etiologies of CVDs but delay in cardiac action potential repolarization is a common mechanism of electrical remodeling (125–127). Most electrophysiological studies suggest that a dip in the K+ currents plays a key role in electrical remodeling (126–129). There is experimental evidence suggesting that alterations in glucose metabolism in cardiomyocytes led to the remodeling of various channels in the ventricle. It will be interesting to learn that how the K+ channel gets altered in DM. Mechanisms behind the upregulation of K^+^ channel activity in cardiomyocytes of patients with DbCM have derailed insulin signaling and glucose utilization. It was shown in the streptozotocin-induced DM murine model that insulin treatment is quite promising in achieving the normal transient outward current (129–131).

In the case of DbCM (induced by type I or type II diabetes), prolonged QT is seen (132–135), increasing the risk of ventricular arrhythmia (136, 137). At the molecular level, this lengthening of the action potential is mainly driven by deregulated expression of various ion channel proteins and their properties (138–140).

Recently, it was found that epigenetic regulators such as miRNAs also participate in myocardial electrical remodeling (141). The expression of voltage-gated potassium channel Kv4.2 is regulated by miR-301a in diabetes (141). Overexpression of miRNA-29 in the diabetic murine model led to structural damage in the heart (142). In another study using a murine model of diabetes, it was seen that an increase in miRNA-141 levels affects ATP production by decreasing mitochondrial phosphate transport (108).

Various studies have underscored the role of HDACs in the regulation of ion channel expression but their exact function still needs to be elucidated. One such study elucidates the regulation of sodium–calcium exchanger (NCX)1 by HDAC5. The NCX1 is involved in Ca^2+^ efflux out of the cells and its expression is regulated by NK2 homeobox 5 (NKX2.5). It is involved in the recruitment of HDAC5 to the NCX gene promoter (143). Another study showed that acetylation of NKX2.5 increases its interaction with HDAC5, whereas deacetylation of NKX2.5 increases its affinity toward the p300 complex (144). Epigenetic regulation of HDACs affects Ca^2+^ flux in cardiomyocytes (145). In this study, the authors reported that the N-terminal of HDAC4 inhibits MEF2 activity, resulting in reduced expression of nuclear orphan receptor NR4A, suppressing the hexamine biosynthetic pathway (145). HDAC2 was downregulated in the porcine model of HF, affecting the potassium channel and prolonging the QT interval (146), leading to inhibition of HDAC2 and affecting the action potential. Inhibition of HDACs using class I inhibitor entinostat is a plausible therapeutic modality for HF that reduces the electrical and structural remodeling in HF (147).

### Histone Deacetylases Inhibitors: A Prospective Therapeutic Modality for DbCM

Histone deacetylases are molecules with pleiotropic function and are involved in crucial homeostatic processes such as proliferation, cell death, and cell cycle. HDAC inhibitors (HDACIs) specifically block Zn^2+^-dependent HDAC enzymes involved in histone acetylation. Recently, the US FDA has approved HDACIs for cancer treatment in clinics (148). Moreover, few reports suggest that regulation of histone acetylation is a promising strategy for the treatment of cardiovascular disease in the preclinical model (149). HDACIs are divided into five categories based on their structure:

A) Hydroxamic acid derivates: (e.g., panobinostat, trichostatin A)

B) Short-chain fatty (aliphatic) acids (e.g., valproic acid (VPA), sodium butyrate)

C) Cyclic peptides (e.g., romidepsin)

D) Benzamides (e.g., entinostat)

E) Sirtuin inhibitors

These HDACIs have been approved by US FDA (150–152). To date, HDACIs are not used in clinical trials for fibrotic diseases, but they have been used in cardiac and lung fibrosis (153–155). The major player in the fibrotic condition is the transition of fibroblast into myofibroblasts (156). There are studies suggesting that HDACIs have reversed myofibroblasts activation in animal models of HF. VPA combated fibrosis in the hypertension murine model by regulating the acetylation of corticoid receptors (157). In the pressure overload mice model, VPA abrogated cardiac remodeling (158). VPA also ameliorated cardiac fibrosis by regulating the ERK1/2 phosphorylation (159). A recent study demonstrated that VPA decreased the remodeling process, therefore leading to the onset of atrial fibrillation (160). Similarly, pan-HDACIs also showed antifibrotic activities, MPT0E014 decreased the expression of Ang II and TGF-β receptors in a murine model of cardiomyopathy (161). Mocetinostat downregulated the expression of HDACs in an HF model mechanistically by increasing apoptosis and reducing the myofibroblast phenotype (162). HDAC6 silencing or inhibition using tubacin reduced the TGF-β1 expression and, thus, decreased cardiac fibrosis (163). There is a need for more comprehensive studies looking into the potential of selective HDACIs for DbCM treatment.

## Conclusion and Future Prospective

**Figure 3:**
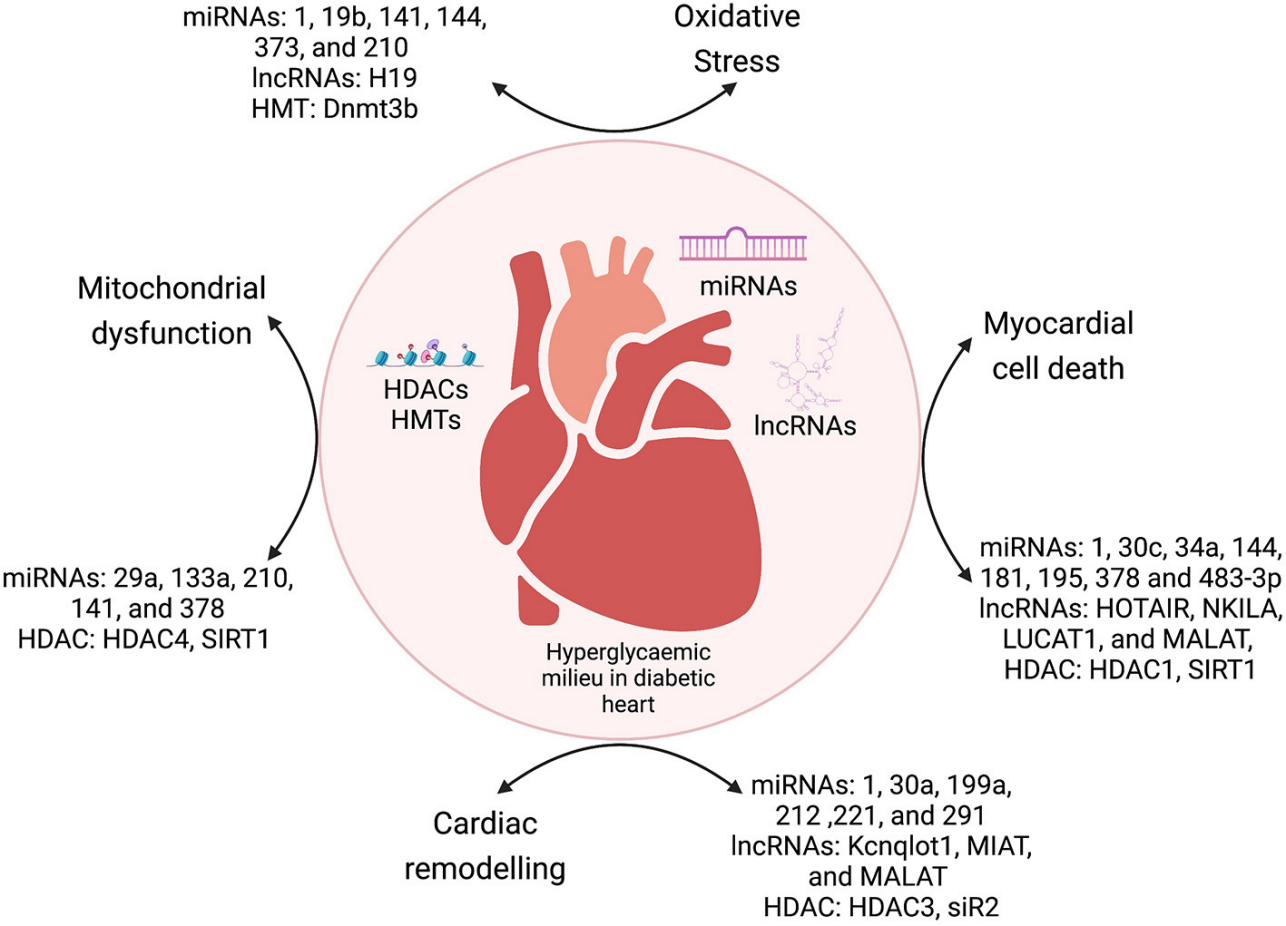
The crosstalk between epigenetic modulators and various mechanisms of DbCM. miRNAs, microRNAs; lncRNAs, long non-coding RNAs; HDACs, histone deacetylases; HMTs, histone methyltransferases; DNMT, DNA methyltransferases.

Diabetic cardiomyopathy is a pleiotropic metabolic disease, with complex etiology and cumulative effects of crosstalk between genetic and epigenetic factors. The diabetic milieu has several inducers of cardiomyopathy such as ROS-mediated oxidative stress, hyperglycemic conditions, cytokines-mediated inflammation, cell death (apoptosis, autophagy, and pyroptosis), and epigenetic regulation of the dysregulated molecular pathways induced by these mediators. Epigenetic modifications range from deregulated ncRNAs (miRNAs and lncRNAs), histone modifications (acetylation and methylation), and DNA promoter methylation, which regulates the expression of important molecules of various pathways mediating DbCM. In summary, previous studies showed that interaction between environmental and genetic factors strongly determine the pathogenesis of DbCM through epigenetic changes in cellular signaling pathways (Figure 3).

The past decade has shown that miRNAs and lncRNAs are important regulators of major molecular pathways such as cell death, oxidative stress, mitochondrial dysfunction, and electrical remodeling (Tables 1, 2). Cardiac fibrosis is an important phenomenon of the cardiac remodeling process in DbCM. There is substantial evidence that epigenetics plays a major role in diabetes-associated cell death. Epigenetic regulatory mechanisms such as histone changes, DNA methylation, miRNAs, and non-coding RNAs regulate cardiac cell death in the diabetic milieu. Similarly, other mechanisms such as mitochondrial dysfunction, oxidative stress, and electrical remodeling are also regulated by miRNAs and by HDACs. The elucidation of these epigenetic mechanisms can provide newer therapeutic strategies for the DbCM. miRNAs and lncRNAs have shown translational potential as diagnostic and prognostic biomarkers and therapeutic modalities for DbCM. It was also shown that HDACs are important regulators in the pathophysiology of DbCM. Inhibition of HDACs using inhibitors has shown promising data in the context of cardiac fibrosis. We have presented a detailed discussion on HDAC inhibitors as promising therapeutic targets for DbCM. However, there is a need to investigate regulatory mechanisms such as chromatin modifications and circular RNAs as contributors to DbCM.

**Table 1:** Deregulated miRNAs in diabetic cardiomyopathy (DbCM).

**miRNAs**	**Process associated**	**Target genes**	**References**
miRNA-212	Cardiac hypertrophy	FOXO3	(35)
miRNA-30c	Cardiac hypertrophy	Cdc42 and Pak1	(36)
miRNA-30c & 181	Cardiac hypertrophy	p53	(38)
miRNA-199a	Cardiac hypertrophy	PGC-1α	(40)
miRNA-200c	Cardiac hypertrophy	DUSP-1	(39)
miRNA-133a	Cardiac fibrosis	TGF-ß1	(43)
miRNA-21	Cardiac fibrosis	DUSP-8	(46)
miRNA-1	Cardiac apoptosis	IGF-1	(62)
miRNA-34a	Cardiac apoptosis	SIRT-1	(77)
miRNA-483-3p	Cardiac apoptosis	IGF-1	(64)
miRNA-195	Cardiac apoptosis	SIRT-1	(65)
miRNA-144	Cardiac apoptosis	IRS	(66)
miRNA-203	Cardiac apoptosis	PIK3CA	(69)
miRNA-532	Cardiac apoptosis	ARC	(70)
miRNA-30c	Cardiac autophagy	Beclin1	(90)
miRNA-30d	Cardiac pyroptosis	Foxo3A	(94)
miRNA-9	Cardiac pyroptosis	ELAVL1	(95)
miRNA-29a	Mitochondrial dysfunction	PPARα	(103)
miRNA-210	Mitochondrial dysfunction	ISCU1/2	(107)
miRNA-141	Mitochondrial dysfunction	SLC25A3	(108)
miRNA-378	Oxidative stress	ATP synthase	(109)
miRNA-144	Oxidative stress	Nrf2	(41)
miRNA-301	Electrical remodeling	Kv4.2	(141)

**Table 2:** Deregulated lncRNAs in DbCM.

**lncRNAs**	**Process associated**	**Target genes**	**References**
MIAT	Hypertrophy; fibrosis	TLR4; TGF-ß1	(49–51)
Kcnq1ot	Fibrosis; pyroptosis	TGF-ß1	(52)
H19	Apoptosis; autophagy	VDAC1; DIRAS3	(73, 92)
MALAT 1	Apoptosis	NLRP3; TGF-ß1; ABCA1	(74–76)
HOTAIR	Apoptosis	SIRT-1	(77)
MEG3	Apoptosis	PDCD 4	(78)
LUCAT1	Apoptosis	CYP11B2	(80)
DCRF	Autophagy	PCDH17	(91)

With the advancement in genomics and molecular biology techniques, such as transposase-accessible chromatin (ATAC) sequencing, deep sequencing, and ChIP-sequencing, high-throughput data on DNA methylomes can be generated. This genome-wide data will provide a comprehensive picture of DbCM. The information, thus, acquired will help to understand the role of epigenetic modulators in DbCM in a pathway-specific manner. This review aims to help in understanding the role of various epigenetic factors in conjunction with specific pathways in DbCM.

## Author Contributions

AM performed the data collection, data approval, manuscript drafting, and manuscript editing. MK performed data collection, manuscript drafting, and manuscript editing. AB performed data manuscript editing. RG performed manuscript editing during revision. All authors contributed to the article and approved the submitted version.

## Conflict of Interest

The authors declare that the research was conducted in the absence of any commercial or financial relationships that could be construed as a potential conflict of interest.

## Publisher's Note

All claims expressed in this article are solely those of the authors and do not necessarily represent those of their affiliated organizations, or those of the publisher, the editors and the reviewers. Any product that may be evaluated in this article, or claim that may be made by its manufacturer, is not guaranteed or endorsed by the publisher.
